# Mortality in a cohort of complex patients with chronic illnesses and multimorbidity: a descriptive longitudinal study

**DOI:** 10.1186/s12904-016-0111-x

**Published:** 2016-04-12

**Authors:** I. Martín-Lesende, E. Recalde, P. Viviane-Wunderling, T. Pinar, F. Borghesi, T. Aguirre, M. Recio, ME Martínez, J. Asua

**Affiliations:** Bilbao-Basurto Integrated Health Organisation, Centro de Salud de San Ignacio Health Centre, C/Larrako Torre, 9, 48015 Bilbao, Spain; Begoña Health Centre. Basque Health Service (Osakidetza), Bilbao, Spain; Basque Office for Health Technology Assessment (OSTEBA), Department of Health, Government of the Basque Country, Vitoria, Spain

**Keywords:** Mortality, Chronic diseases, Heart failure, Chronic lung disease, Elderly, Telemonitoring, Primary care, Hospital admissions

## Abstract

**Background:**

Certain advanced chronic conditions (heart failure, chronic lung disease) are associated with high mortality. Nevertheless, most of the time, patients with these conditions are not given the same level of attention or palliative care as those with cancer.

The objective of this study was to assess mortality and its association with other variables in a cohort of complex multimorbid patients with heart failure and/or lung disease from two consecutive telemonitoring studies.

**Methods:**

This multicentre longitudinal study was conducted between 2010 and 2015. We included 83 patients (27 without telemonitoring) with heart failure and/or lung disease with > 1 hospital admission in the previous year and great difficulties leaving home or were housebound. The following variables were indicators of their complex clinical condition: old age (mean: 81 years), comorbidity (Charlson Comorbidity Index score ≥ 2: 86.2 %), both conditions concurrently (54.2 %) and home oxygen therapy (52 %).

We assessed mortality (rate, cause and place of death) and its association with: age, sex, telemonitoring, functional status (Barthel score), quality of life (EQ-5D visual analogue scale), number of medications, and all-cause and condition-specific (due to conditions prompting inclusion) admissions during the previous year. Uni- and bivariate analysis and logistic regression were performed, considering *p <* 0.05 significant.

**Results:**

A total of 61 patients died within 5 years, representing 31.2 %/year (95 % CI: 23–40.1 %), considering the overall follow-up (sum of individual follow-up days). Of these, 81 % of deaths (95 % CI: 69.1–89–1 %) were due to the condition prompting inclusion, and 83.3 % (95 % CI: 72–90.7 %) died in hospital (median: 8.5 days).

Mortality was lower among those under telemonitoring (*p =* 0.027), and with fewer condition-specific admissions the previous year (*p =* 0.006); the latter also showed the strongest association in the multivariate analysis (Exp(B) = 6.115).

**Conclusions:**

Complex patients with multimorbidity had a high mortality rate, generally dying due to the condition for which they had been included, and in hospital (83.3 %). New approaches for managing such patients should be considered, introducing palliative care as required, and using more comprehensive predictors of mortality (functional status and quality of life), together with those related to the illness itself (previous admissions, progression and symptoms).

## Background

When we consider end-of-life patients under palliative care, there is a tendency to think of patients with terminal cancer; indeed, in practice, palliative care is mainly focused on this type of patient. However, given current demographic changes, with progressive population ageing and longer survival, advanced chronic illnesses are becoming the leading cause of patients requiring end-of-life care. At the same time, we should consider that the palliative care model involves starting to take certain measures well before advanced stages of illness leading towards death. Ideally, palliative care should overlap with curative treatments in cases of poor prognosis, this being considered a more comprehensive patient-centred approach, taking into account not only clinical but also psychosocial and existential needs. Its objective is to avoid discomfort and suffering in the wider sense, improving quality of life as much as possible and avoiding unnecessary treatments.

The two best examples of this situation are chronic obstructive pulmonary disease (COPD) and heart failure (HF), due to their prevalence and the percentage of patients in final and irreversible stages. However, as we will discuss below, it is difficult to define the terminal state, and the most common scenario is that such patients are not given palliative care, but rather continue on care pathways designed for earlier stages of the illness.

COPD is characterised by progressive deterioration over time, with persistence of symptoms, episodes of exacerbation, and frequent hospitalisation, as well as a high mortality rate in the most advanced patients, of around 30 % per year [1–3]. Despite this, relatively few patients receive standard palliative care [4], only 5 % according to some authors [5], and they even have limited access to general healthcare in these advanced stages of the disease, when they spend most of their time at home. It is not easy to establish when their shortened life expectancy makes such patients candidates for receiving mainly palliative care. However, there are specific tools, such as the Body mass index, airflow Obstruction, Dyspnoea, and Exercise (BODE) index, and certain factors such as the degree of deterioration of pulmonary function, frequency of exacerbations and hospital admissions, and progression of respiratory failure (dyspnoea, need for continuous oxygen therapy, etc.) that can guide us on when to initiate this type of care [6–8].

HF shares many characteristics with COPD in terms of progression and prognosis. There is also progressive deterioration, in advanced stages of the illness, it is associated with frequent exacerbations and hospitalisation, and in the end stages death is often predictable. However, it is not uncommon that patients receive aggressive therapies until death despite their poor prognosis [9]. As for COPD, there are tools to assess who might benefit from palliative care, such as the CARING (Cancer, Admissions ≥ 2, Residence in a nursing home, Intensive care unit admission with multiorgan failure, ≥ 2 Non-cancer hospice Guidelines) criteria and criteria based on National Hospice Organization guidelines, for assessing shortened life expectancy, although such an assessment can also be based on recurrent and evolving signs and symptoms [9]. In cases of severe HF, the mortality rate is also around 30 % per year.

Despite it being common for older adults to have one or more chronic conditions at advance stages, these conditions coexisting and interacting with the individual’s functional and overall health status, very few studies have addressed mortality and palliative care by considering multimorbidity in a comprehensive manner.

The consecutive studies TELBIL and TELBIL-A consisted of a clinical trial and an implementation evaluation, respectively [10–12], of primary care-based telemonitoring of chronic patients. These studies have had two main characteristics; on the one hand, the importance of primary care in the management of the telemonitoring and follow-up of patients, and on the other, the multimorbidity and complexity of the patients (with chronic lung disease and/or HF). For this reason, they represent an opportunity for analysing mortality, its characteristics and implications, in a comprehensive manner with a focus on multimorbidity.

The objective of this study was to describe the characteristics of mortality and its association with other relevant variables, in a cohort of complex multimorbid patients with HF and/or chronic lung disease (in most cases, COPD) from the TELBIL and TELBIL-A studies, regardless of whether they had been under telemonitoring.

## Methods

### Study design and location

**Fig. 1:**
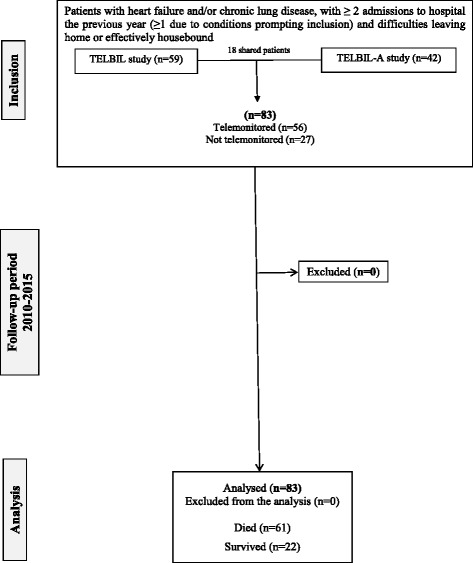
Study design and patient flow

Multicentre descriptive longitudinal study of the cohort of all the patients who participated in two consecutive and overlapping telemonitoring studies (TELBIL and TELBIL-A) from February 2010 to February 2015, the overall period during which patients were followed up. Figure 1 shows the flow of patients through the study.

The research was conducted in the urban Bilbao-Basurto Integrated Healthcare Organisation. This organisation is composed of a reference hospital (Basurto University Hospital) and 25 health centres (with a combined staff of 230 general practitioners, 320 nurses and 72 paediatricians), and a catchment population of 382,000 people, of whom over 20 % were ≥ 65 years old. Patients in the area can also be referred to Santa Marina hospital, a subacute hospital, focused on mainly caring for patients with multimorbidity with their own palliative care unit.

### Study patients

In the analysis, we included all the patients from both studies (TELBIL and TELBIL-A, *n =* 83) whether they had been under telemonitoring (56) or not (27) during the study period; 18 patients had been included in both studies. The patients were all complex patients with multimorbidity, as a result of the inclusion criteria applied, especially the requirement that they had great difficulties leaving their home or were effectively housebound, this implying very poor baseline functional status. Table 1 summarises the most relevant characteristics that define this complexity.

**Table 1:** Main characteristics characterising the complexity of the patients included in the TELBIL and TELBIL-A studies

Variable	Value^a^
Age, years	81.3 (IQR: 77.1 to 85.4)
Condition prompting inclusion	Heart failure 21.7 %; chronic lung disease 24.1 %; both concurrently 54.2 %
Severity of the COPD^b^ based on FEV1^c^	82.6 % severe or very severe
Charlson Comorbidity Index score ≥ 2^c^	86.2 %
Number of different regular medications	11 (IQR: 9 to 14)
Continuous home oxygen therapy^c^	51.7 %
BADL^d^, Barthel Index score on inclusion	75 (IQR: 50 to 90)
HRQoL^e^, EQ-5D VAS score^e^ on inclusion	40 (IQR: 30 to 60)
Lack of social support (specific questionnaire)^c^	17.2 %
Number of all-cause admissions the year before inclusion	3 (IQR: 2 to 4)
Number of condition-specific admissions^f^ the year before inclusion	2 (IQR: 1 to 3)
Number of home visits by a doctor and/or nurse the year before inclusion^c^	22.5 (IQR: 12 to 31)

^a^Results are expressed as percentages, if they are qualitative variables, or mean and interquartile range (IQR), if they are quantitative

^b^COPD: chronic obstructive pulmonary disease

^c^Data for the sample of 59 patients from the TELBIL study (randomised clinical trial); the rest refer to all 83 patients

^d^BADL: basic activities of daily living

^e^HRQoL: health-related quality of life; EQ-5D VAS: EuroQol EQ-5D visual analogue scale

^f^Only hospital admissions due to the conditions (heart failure and/or chronic lung disease) that prompted inclusion are considered

### Inclusion criteria

In both projects, we included:

- adult patients with HF and/or chronic lung disease (COPD, chronic asthma, or others)
- with two or more admissions to hospital in the previous year (at least one of these being due to the condition that prompted inclusion in the study)
- who had great difficulties leaving their home or were effectively housebound, meaning that they were usually provided care at home.

### Exclusion criteria

We excluded patients in long-term care, with a life expectancy of less than 6 months due to an illness other than those related to their inclusion in the study, with potential barriers to continuous follow-up, or social/family circumstances that might have hindered their participation.

### Ethical approval

Both studies (TELBIL and TELBIL-A) were approved by the corresponding clinical research ethics committee (that of Basurto University Hospital) and the primary care research committee. Before patients were included in the study, they or their relatives provided written informed consent.

### Study variables

The primary variable in this study was mortality, including its rate and other characteristics:

- cause of death, and whether it was due to a condition that prompted inclusion (condition-specific mortality),
- place of death (in hospital or at home) and duration of hospital stay until death, in such cases.

We also collected data on:

- patient age in years on inclusion in the study and sex
- health conditions for which patients were included (HF, chronic lung disease, or both)
- whether they were under telemonitoring during some of the follow-up period
- assessment of ability to perform basic activities of daily living (BADL), the Barthel Index score [13] on inclusion
- assessment of health-related quality of life (HRQoL) (EuroQol EQ-5D) [14] on inclusion
- number of medications taken on a regular basis on inclusion
- number of admissions to hospital in the year prior to inclusion, considering both all-cause and condition-specific admissions.

### Estimation of the power of the study

We included all patients who participated in the two previous studies (TELBIL and TELBIL-A), and hence, sample size calculations were carried out prior to this mortality analysis, with estimates being based on hospital admissions. However, considering a prevalence of 300 patients with these characteristics in the catchment population (based on the lists used for the initial recruitment), and the 83 patients included, and assuming a mortality of 30 % and a margin of error of 5 %, the power of the study would be 80 %.

#### Source of data

Data were collected from the primary care electronic health records and hospital electronic discharge records, as well as from the databases created for each of the TELBIL studies.

### Data analysis

We carried out a univariate analysis of the variables, using the mean and the standard deviation, or the median and the interquartile range (IQR), as measures of central tendency and dispersion, as appropriate, depending on whether data were normally distributed. We estimated population values, inferential statistics, for the most important values (rate and place of death). To assess the association between mortality and the rest of variables studied, bivariate analysis was performed with Student’s *t* test or Mann–Whitney *U* test (in the case of non-normally distributed or ordinal data) for the quantitative variables, and the chi-squared test for qualitative variables. The analysis was completed with a multivariate binary logistic regression analysis, using the “Enter” method, considering whether a patient had died during the study period (nominal variable) as the dependant variable.

All the analyses were carried out using the IBM SPSS Statistics for Windows version, 20. The level of statistical significance was set at *p <* 0.05.

## Results

We included 83 patients of whom 35 were female (42.2 %) and the median age of the sample on inclusion was 81.6 years (IQR: 77.1 to 85.4; range 51 to 95). The conditions prompting inclusion were HF in 21.7 % of patients (18), and chronic lung disease in 24.1 % (20) (the majority of these patients, 78.6 %, having COPD), while 52.4 % (45) had both diseases at the same time. A total of 56 patients were telemonitored (67.5 %), some for the full 5 years of follow-up, the other 27 patients being in the control group in the TELBIL study.

During the study period, 61 patients died (73.5 %, 95 % CI 63–82 %). Considering an overall follow-up of 190 patients/year, based on a cumulative total of 69,380 days of follow-up for the 83 patients, this corresponds to a mortality rate of 31.2 % patients/year (95 % CI: 23–40.1 %).

Regarding causes of death, 81 % (95 % CI 69.1 %–89.1 %) died due to the condition that had prompted their inclusion (condition-specific mortality). Overall, 34.5 % died due to a heart condition (all of them due to HF) and 48.3 % due to a respiratory condition (of these, 57.1 % dying due to respiratory infection or pneumonia, 39.3 % due to exacerbation of lung disease or respiratory failure without infection, and 1 patient due to an associated lung tumour); while in 3 cases it was not possible to determine the cause of death. Ten patients died at home (16.7 %, 95 % CI: 9.3–28 %), and 50 in hospital (83.3 %, 95 % CI: 72–90.7 %), 4 of these dying in the emergency department, while in 1 patient it was not possible to determine the place of death. Out of the 46 patients who died during admission, the median length of hospital stay was 8.5 days (IQR: 4 to 15.3, with a maximum value of 35 days).

Table 2 shows the relationship between whether patients died and the rest of the study variables. We found the mortality rate was significantly lower among those who had been under telemonitoring, and significantly higher among those with more condition-specific admissions in the year prior to inclusion.

**Table 2:** Association between whether or not patients from the TELBIL and TELBIL-A studies died during follow-up and the other variables

	Died (*n =* 61)	Survived (*n =* 22)	*p* value^a^
Telemonitored during study period^b^			**0.027**
Yes, n (%)	37 (60.7 %)	19 (86.4 %)	
No, n (%)	24 (39.3 %)	3 (13.6 %)	
Condition prompting inclusion			0.888
Lung disease (78.6 % COPD)	14 (22.9 %)	6 (27.3 %)	
Heart failure	13 (21.3 %)	5 (22.7 %)	
Both concurrently	34 (55.8 %)	11 (50 %)	
Sex			0.889
Female	26 (42.6 %)	9 (40.9 %)	
Male	35 (57.4 %)	13 (59.1 %)	
Age in years on inclusion, median (IQR)	81 (78 to 87)	81.3 (71 to 84)	0.192
Barthel index score (0 to 100)	70 (40 to 87.5)	87.5 (60 to 100)	0.120
EQ-5D VAS score^c^ (0 to 100)	40 (25 to 60)	50 (37.5 to 65)	0.148
Number of regular medications	11 (9 to 13)	12 (10 to 14)	0.219
Number of all-cause admissions the year before inclusion	3 (2 to 4)	3 (1 to 4)	0.384
Number of condition-specific (heart or lung-related) admissions the year before inclusion	2 (2 to 3)	2 (1 to 2)	**0.006**

^a^For the qualitative variables (having been telemonitored or not, inclusion condition and sex, expressed as frequencies and percentages), we used chi-square for hypothesis testing. For the others, qualitative variables (expressed as medians and interquartile ranges [IQRs]), we used the non-parametric Mann–Whitney *U* test, as the data were not normally distributed or the sample size was small

^b^In complementary analysis to assess the influence of the length of follow-up, the difference in follow-up time between those who were and were not telemonitored (mean of 851 vs. 805 days respectively) was not found to be significant (*p =* 0.712)

^c^EQ-5D VAS: EuroQol EQ-5D visual analogue scale

in bold variables con statistical significance <0.05

We carried out multivariate logistic regression analysis, considering whether the patient died as the dependent variable, and the rest of the variables as the independent variables. The omnibus chi-square value was <0.0001 (indicating that the model explains the event), and the overall percentage who were correctly classified was 81.8 %, indicating that the model was explanatory. Table 3 summarises the results of this analysis, using the Enter method. We found that the following variables were significant for explaining the dependent variable: age, Barthel Index score, and EQ-5D visual analogue scale score on inclusion; and all-cause (a protective effect) and condition-specific admissions. The variable most closely related to dying was the number of condition-specific admissions in the previous year.

**Table 3:** Statistics for the independent variables from the logistic regression analysis, using the Enter method, considering whether a patient had died as the dependent variable

	B	Standard error	Wald	Degrees of freedom	Statistical significance	Exp(B)
Telemonitoring	–1.622	.971	2.789	1	.095	.197
Inclusion condition	.248	.470	.278	1	.598	1.281
Sex	–.228	.802	.081	1	.776	.796
Age in years on inclusion	.107	.053	3.990	1	**.046**	1.113
Barthel Index score (BADL)^a^	–.041	.020	4.179	1	**.041**	.959
EQ-5D VAS score (HRQoL)^b^	–.049	.024	4.185	1	**.041**	.952
Number of regular medications	.030	.124	.060	1	.807	1.031
Number of all-cause admissions the year before inclusion	–1.278	.424	9.085	1	**.003**	.278
Number of condition-specific admissions^c^ the year before inclusion	1.811	.537	11.356	1	**.001**	6.115
Constant	–1.551	4.615	.113	1	.737	.212

^a^BADL: basic activities of daily living

^b^EQ-5D VAS: EuroQol EQ-5D visual analogue scale; HRQoL: health-related quality of life

^c^Only hospital admissions due to the conditions (heart failure and/or chronic lung disease) that prompted inclusion are considered

in bold variables con statistical significance <0.05

## Discussion

The baseline characteristics of the patients included in the TELBIL and TELBIL-A studies, including concurrent health problems with 50 % having both HF and lung disease, reflect the high level of comorbidity and complexity of these patients. This gave us the opportunity to conduct research considering their complexity and overall characteristics, rather than focusing on a specific pathology or health condition, which has previously been the usual approach.

The TELBIL telemonitoring strategy has shown to decrease the number of admissions and length of hospital stay, with good cost-effectiveness ratios, and good acceptance among patients and their families as well as health professionals [11, 12]. This was achieved in patients who in many cases had a poor health status and short life expectancy. Building on the previous TELBIL studies, we have carried out a specific analysis of mortality, and the results can contribute to the identification of new strategies to help improve the wellbeing of these kinds of patients and their families.

Our patients had a high mortality rate, estimated to be 31.2 %/year, which is consistent with figures reported by other authors [1–3, 7, 15, 16]. In our case, 73.5 % of patients died within the 5-year study period, although few patients had been followed-up for the entire period (open inclusion). This result underlines the high complexity and advanced stage of illness in our patients, as the rate is somewhat higher than that observed by other authors over 5 full years of follow-up of patients with advanced COPD, mortality over the follow-up period being around 70 % [3, 6].

The majority of deaths were due to health problems that prompted inclusion in the study (HF and lung disease), condition-specific mortality, as could be expected. In patients whose inclusion was based on them having HF, all cases of death due to heart-related causes were associated with decompensation of the HF, sometimes triggered by other factors. In those whose inclusion was based on them having lung disease, nearly all deaths were due to exacerbation of the disease, in some cases associated with a respiratory infection. That is, our patients died due to the health conditions that had prompted their inclusion and to a limited range of associated causes. This specificity and association of death with patients’ primary health problems has also been described by other authors [17–19].

Taking into account the aforementioned factors (complexity and severity of patients’ condition, high mortality, specificity and predictability of the causes of death), the most striking finding in our study was that the majority of patients (83.3 %) died in hospital rather than at home. This pattern has also been reported by other authors [20], and is despite the fact that their own home may be the most comfortable and appropriate place to die, and according to the literature, the place where a high percentage of this type of patient say they would prefer to die [7, 21, 22]. Based on the review of the clinical records (not supported with specific analysis), we appreciated that during the hospitalization in which the patients died, treatment and management did not differ generally from others the patients had previously except, in many cases, in the palliative care once near death. This seems to indicate that we continue to focus on the illness and have not taken on board the concept of palliative care, that we do not have the resources to make it easier for these patients stay in their homes in the end stages of their illness, or more likely, a combination of both. Further, we should also consider the length of hospital stays during which patients died, with a median of 8.5 days and more than15 days in 25 % of cases, recognising that this period is likely to have been highly burdensome given the characteristics of the conditions and commonly associated symptoms (e.g., severe dyspnoea).

The number of condition-specific admissions (attributable to the conditions that prompted inclusion) in the previous year was found to be strongly associated with mortality in both the bivariate and multivariate models. As we have mentioned earlier, hospitalisation is frequent in the advanced stages of these illnesses, and behaves as a predictive factor. In the multivariate analysis, other variables that were found to be associated with mortality were functional status (Barthel Index score) and perceived quality of life (EQ-5D visual analogue scale score), and consideration of these two factors is likely to be key to the proper management of these patients on the basis of their multimorbidity and complexity rather than specific illnesses. In fact, functional status was found to be the single most important factor in the prediction of in-hospital death, above other measures of illness severity [9]. Further, the new technologies for supporting the provision of care (telemonitoring) have also been found to have an effect, especially in reducing hospitalisation [11, 12], and tending to delay death, although the latter finding was not conclusive, largely due to the small sample size. The result obtained in terms of all-cause hospital admissions in the previous year was unclear, in that it seemed to indicate, according to the logistic regression, that the more all-cause admissions, the lower the mortality. This finding must be interpreted with care, however, as it is inconsistent with the bivariate analysis, in which admissions in the previous year was strongly positively associated with mortality, helping to explain death in a more rational way, and further this admission rate is probably linked to other variables studied.

The community setting, in particular primary care, is an important environment in which to monitor and manage this type of complex patient, given its accessible location and the approachability of the health care providers, frequency of contacts, and efficiency. It is important to have sufficient professional training and resources, including nurse case managers, who, although they seem to have limited effect on readmissions, do seem to have a positive impact on patient quality of life and satisfaction with care provided [23], and palliative care teams [4]. As well as ensuring good coordination between the different levels of care and services [7, 24], patients should be involved in their own care, progression and prognosis of their conditions, and their preferences about care should be considered, steps which do not seem to be taken in most cases [22, 25]. We consider there is a progressive improvement in the community health site in all these aspects, including the support by caregivers and relatives, although there is still much to be done, with the main target of giving the best and most comfortable assistance.

We believe that is important to start to consolidate the management of these patients in a different way:

- considering functional status, history of hospitalisation, HRQoL and comorbidity, to have a better idea of the overall health status and life expectancy of complex multimorbid patients and specifically those with HF or chronic lung disease
- assessing life expectancy, to determine the type of care that is appropriate, and starting palliative care when required, even overlapping with curative care at earlier stages of illness
- considering the use or the need to make available specific complementary healthcare resources and support to facilitate better management of these patients, with appropriate coordination between levels of care and involving patients, and their relatives and caregivers, in patient care.

The fact that we selected highly-complex patients for this research, particularly by applying the inclusion criterion of having great difficulties leaving home or being effectively housebound, may hinder the generalisation of our findings to other less extreme situations. However, we believe that this limitation is relatively unimportant for the interpretation and discussion of the results, given the agreement we find when comparing our findings with those of other studies.

It is necessary to continue searching for strategies to help characterise terminal status in non-cancer patients, and assessing new strategies for managing such patients.

## Conclusions

Complex multimorbid patients with HF or lung disease often have a short life expectancy, and despite this, in most cases, no consideration is given to providing adequate care based on a palliative care model. As a consequence, most such patients die in hospital, many after relatively long stays, which are likely to have been highly burdensome, far from the social and family environment that would be more suitable (in particular, their own home).

There is need for another approach to this situation and the management of these patients, based on predictive factors, such as functional status, that are more comprehensive than those associated with their specific pathologies or health conditions, and considering quality of life, as well as a coordinated and comprehensive healthcare system with sufficient resources, with the community/primary care setting playing a leading role.

Nevertheless, a history of hospitalisation due to the primary illness, and intensity and progression of associated symptoms, signs and test results may help to assess the life expectancy of these patients.

## References

[1] Hosker HSR, Anstey K, Lowe D, Pearson M, Roberts MC (2005). The outcome of patients with COPD admitted to UK hospitals with an exacerbation. Results from the RCP/BTS national COPD audit. Eur Respir J.

[2] Groenewegen KH, Schols AM, Wouters EF (2003). Mortality and mortality related factors after hospitalization for acute exacerbation of COPD. Chest.

[3] Soriano JB, Maier WC, Egger P, Visick G, Thakrar B, Sykes J (2000). Recent trends in physician diagnosed COPD in women and men in the UK. Thorax.

[4] Escarrabill J, Soler-Cataluña JJ, Hernández C, Servera E (2009). Recommendations for End-of-Life Care in Patients With Chronic Obstructive Pulmonary Disease. Arch Bronconeumol.

[5] Goodridge D, Lawson J, Duggleby W, Marciniuk D, Rennie D, Stang M (2008). Health care utilization of patients with chronic obstructive pulmonary disease and lung cancer in the last 12 months of life. Respir Med.

[6] O’Donnell DE, Hernández P, Kaplan A, Aaron S, Bourbeau J, Marciniuk D (2008). Canadian Thoracic Society recommendations for management of chronic obstructive pulmonary disease—2008 update- highlights for primary care. Can Respir J.

[7] Seamark D, Seamark CJ, Halpin DMG (2007). Palliative care in chronic obstructive pulmonary disease: a review for clinicians. J R Soc Med.

[8] Lanken PN, Terry PB, Delisser HM, Fahy BF, Hansen-Flaschen JH, Heffner JE (2008). An official American Thoracic Society clinical policy statement: palliative care for patients with respiratory diseases and critical illnesses. Am J Respir Crit Care Med.

[9] Martínez-Sellés M, Vidánb MT, López-Palopc R, Rexachd L, Sánchez E, Datino T (2009). El anciano con cardiopatía terminal. Rev Esp Cardiol.

[10] Martín-Lesende I, Orruño E, Cairo MC, Bilbao A, Asua J, Romo MI (2011). Assessment of a primary care-based telemonitoring intervention for home care patients with heart failure and chronic lung disease. The TELBIL study. BMC Health Services Research.

[11] Martín-Lesende I, Orruño E, Bilbao A, Vergara I, Cairo MC, Bayón JC (2013). Impact of telemonitoring home care patients with heart failure or chronic lung disease from primary care on healthcare resource use (the TELBIL study randomised controlled trial). BMC Health Serv Res.

[12] Orruño E, Martín-Lesende I, Mateos M, Recalde E, Reviriego E, Bayón JC, Asua J (2014). Telemonitorización de Pacientes Pluripatológicos con Enfermedad Cardiaca o Respiratoria. Evaluación de su Implantación en Atención Primaria. Spanish Ministry of Health, Social Services and Equality. Basque Office for Health Technology Assessment (OSTEBA).

[13] Mahoney FI, Barthel DW (1965). Functional evaluation: the Barthel Index. Md Med J.

[14] Oppe M, Rabin R, de Charro F (2007). EQ-5D User Guide. EuroQol Group. Version 1.0.

[15] Almagro P, Calbo E, Ochoa de Echaguen A, Barreiro B, Quintana S, Heredia JL, Garau J (2002). Mortality after hospitalization for COPD. Chest.

[16] Formiga F, Manitob N, Pujola R (2007). Insuficiencia cardiaca terminal. Med Clin (Barc).

[17] Zielinski J, MacNee W, Wedzicha J, Ambrosino N, Braghiroli A, Dolensky J (1997). Causes of death in patients with COPD and chronic respiratory failure. Monaldi Arch Chest Dis.

[18] Vilkman S, Keistinen T, Tuuponen T, Kivela SL (1997). Survival and cause of death among elderly chronic obstructive pulmonary disease patients after first admission to hospital. Respiration.

[19] Nishimura K, Tsukino M (2000). Clinical course and prognosis of patients with chronic obstructive pulmonary disease. Curr Opin Pulm Med.

[20] Fried TR, Pollack DM, Drickamen MA, Tinetti ME (1999). Who dies at home? Determinants of site of death for community-based long term care patients. J Am Geriatr Soc.

[21] Higginson IJ, Sen-Gupta GJ (2000). Place of care in advanced cancer: a qualitative systematic literature review of patient preferences. J Palliat Med.

[22] Gore JM, Brophy CJ, Greenstone MA (2000). How well do we care for patients with end stage chronic obstructive pulmonary disease (COPD)? A comparison of palliative care and quality of life in COPD and lung cancer. Thorax.

[23] Hermiz O, Comino E, Marks G, Daffurn K, Wilson S, Harris M (2002). Randomised controlled trial of home based care of patients with chronic obstructive pulmonary disease. BMJ.

[24] Mitchell G, Zhang J, Burridge L, Senior H, Miller E, Young S (2014). Case conferences between general practitioners and specialist teams to plan end of life care of people with end stage heart failure and lung disease: an exploratory pilot study. BMC Palliative Care.

[25] Elkington H, White P, Higgs R, Pettinari CJ (2001). GPs’ views of discussions of prognosis in severe COPD. Fam Pract.

